# Phenotyping Flowering in Canola (*Brassica napus* L.) and Estimating Seed Yield Using an Unmanned Aerial Vehicle-Based Imagery

**DOI:** 10.3389/fpls.2021.686332

**Published:** 2021-06-17

**Authors:** Ti Zhang, Sally Vail, Hema S. N. Duddu, Isobel A. P. Parkin, Xulin Guo, Eric N. Johnson, Steven J. Shirtliffe

**Affiliations:** ^1^Department of Plant Sciences, College of Agriculture and Bioresources, University of Saskatchewan, Saskatoon, SK, Canada; ^2^Saskatoon Research and Development Center, Agriculture and Agri-Food Canada, Saskatoon, SK, Canada; ^3^Department of Geography and Planning, University of Saskatchewan, Saskatoon, SK, Canada

**Keywords:** canola, flowering, seed yield, multispectral camera, remote sensing

## Abstract

Phenotyping crop performance is critical for line selection and variety development in plant breeding. Canola (*Brassica napus* L.) flowers, the bright yellow flowers, indeterminately increase over a protracted period. Flower production of canola plays an important role in yield determination. Yellowness of canola petals may be a critical reflectance signal and a good predictor of pod number and, therefore, seed yield. However, quantifying flowering based on traditional visual scales is subjective, time-consuming, and labor-consuming. Recent developments in phenotyping technologies using Unmanned Aerial Vehicles (UAVs) make it possible to effectively capture crop information and to predict crop yield *via* imagery. Our objectives were to investigate the application of vegetation indices in estimating canola flower numbers and to develop a descriptive model of canola seed yield. Fifty-six diverse *Brassica* genotypes, including 53 *B. napus* lines, two *Brassica carinata* lines, and a *Brassica juncea* variety, were grown near Saskatoon, SK, Canada from 2016 to 2018 and near Melfort and Scott, SK, Canada in 2017. Aerial imagery with geometric and radiometric corrections was collected through the flowering stage using a UAV mounted with a multispectral camera. We found that the normalized difference yellowness index (NDYI) was a useful vegetation index for representing canola yellowness, which is related to canola flowering intensity during the full flowering stage. However, the flowering pixel number estimated by the thresholding method improved the ability of NDYI to detect yellow flowers with coefficient of determination (*R*^2^) ranging from 0.54 to 0.95. Moreover, compared with using a single image date, the NDYI-based flowering pixel numbers integrated over time covers more growth information and can be a good predictor of pod number and thus, canola yield with *R*^2^ up to 0.42. These results indicate that NDYI-based flowering pixel numbers can perform well in estimating flowering intensity. Integrated flowering intensity extracted from imagery over time can be a potential phenotype associated with canola seed yield.

## Introduction

Canola (*Brassica napus* L.) is the predominant oilseed crop grown in Canada (Clayton et al., 2000). Canada has the largest area of canola production in the world (Statistics Canada, 2018). With the growing global demand for canola, Canada needs to maintain and improve canola yield and seed quality to meet the market demands. Yield components of canola consist of the number of pods, the seeds per pod, and the weight per seed (Tayo and Morgan, 1975; McGregor, 1981; Diepenbrock, 2000; Ivanovska et al., 2007; Faraji, 2012). Among these components, pod number retained at maturity is the most important factor as it is influenced most by environmental constraints (Tayo and Morgan, 1975; McGregor, 1981; Diepenbrock, 2000; Ivanovska et al., 2007; Faraji, 2012; Gan et al., 2016; Kirkegaard et al., 2018). The flowering stage in canola is important for yield estimation as flowers produced in the first 2–3 weeks from anthesis contribute to 75% of the pods at maturation (Tayo and Morgan, 1975). Additionally, the flowering period can last from 2 to 6 weeks, which is a major portion of the crop growth cycle (Gan et al., 2016; Kirkegaard et al., 2018). Thus, flower production is one of the most important factors in determining final seed yield (Tayo and Morgan, 1975; Diepenbrock, 2000; Faraji et al., 2008; Faraji, 2012; Fang et al., 2016; Gong et al., 2018; Kirkegaard et al., 2018; Zhang and Flottmann, 2018).

During the plant breeding process, field-based phenotyping plays an important role in evaluating plant performance. It contributes to the selection of ideal genotypes that are high-yielding by associating genotype with the corresponding phenotype (Montes et al., 2007; Sankaran et al., 2015). To select better canola lines and eventually develop better varieties, breeders need to assess many distinct lines grown in multiple environments to detect interactions between genotype and environment (White et al., 2012; Araus and Cairns, 2014).

The quantification of flowering intensity based on traditional visual scales is subjective, labor-consuming, and is often destructive (Sulik and Long, 2015; Fang et al., 2016; Wan et al., 2018). Although ground-based platforms such as Greenseeker, Crop Circle, or time-lapse RGB imaging can provide adequate spectral data, these platforms still require a prohibitive amount of time and labor (Xu et al., 2018; Hassan et al., 2019). Additionally, data collection using these ground-based platforms may cause soil compaction and crop canopy damage (Xu et al., 2018). Therefore, it is necessary to develop an objective, non-destructive, and efficient method to estimate flower numbers. With this, one can model seed yield by assessing real-time radiometric data of the crop canopy, which has the potential to accelerate breeding methods for yield improvement. Current improvements in aerial-based platforms and sensors equipped on aerial platforms make it possible to effectively collect phenotypes *via* analyzing digital imagery (Kim et al., 2019). Unmanned aerial vehicles (UAVs) equipped with various sensors can quickly provide large quantities of field data enabling plant breeders to efficiently detect traits of numerous plots in large-scale field trials (Kefauver et al., 2017).

Spectral reflectance of the crop canopy is strongly correlated with morphological and physiological traits. Leaf composition and molecular structure can affect the reflectance of the crop; thus, ratios or differences of different bands in the visual light, near IR (NIR), and shortwave IR wavelengths (i.e., vegetation indices) can be a tool to characterize plant traits (Sankaran et al., 2015; Wójtowicz et al., 2016). Previous studies have shown that multispectral reflectance profiles of visible bands (i.e., blue, green, and red) and NIR bands could estimate canopy features, such as nitrogen use efficiency (Kefauver et al., 2017; Prey et al., 2020), leaf area index (Tunca et al., 2018; Blancon et al., 2019), and flower numbers (Guo et al., 2015; Sulik and Long, 2015, 2016; Carl et al., 2017; Gong et al., 2018; Wan et al., 2018; Xu et al., 2018). These plant traits investigated remotely have the potential to improve yield estimates. Flower numbers, as an important factor in determining crop yield, have exhibited close correlations with optical properties in various crops, such as rice (Guo et al., 2015), cotton (Xu et al., 2018), and canola (Sulik and Long, 2015, 2016; Gong et al., 2018; Wan et al., 2018). Guo et al. (2015) applied a machine learning model, the support vector machine, for flowering quantification using RGB images in rice, which resulted in a good correlation between the actual rice flowering panicles and identified flowering (correlation coefficients ranging from 0.64 to 0.82) (Guo et al., 2015). In canola, there are three different canopy morphologies during the growing season, namely, the vegetative phase (green canopy dominated by leaves), the flowering phase (yellow canopy dominated by the yellowness of flower petals), and the mature phase (green or brown canopy because of pods and branches) (Sulik and Long, 2016). During the flowering phase, the yellowness of canola petals is due to carotenoid absorption of blue and reflectance of a mixture of green and red wavelengths (Sulik and Long, 2015, 2016), but the yellow color has little impact on red edge and NIR reflectance unlike a green vegetative canopy (Shen et al., 2009; Migdall et al., 2010; Sulik and Long, 2015, 2016). Thus, the contributed red light decreases the normalized difference vegetation index (NDVI) values (Equation 1) and adversely impact the ability of NDVI to monitor crop growth condition and estimate yield during the flowering phase (Shen et al., 2009, 2010; Sulik and Long, 2015, 2016). Sulik and Long (2015) found that the ratio of green and blue was strongly correlated with the actual flower numbers with a coefficient of determination (*R*^2^) of 0.87, and they proposed the plot-level normalized difference yellowness index (NDYI) (Equation 2) could be a potential yield predictor (*R*^2^ = 0.76) (Sulik and Long, 2016). d'Andrimont et al. (2020) and Han et al. (2021) reported that NDYI successfully captured canola yellowness and detected the peak flowering dates using Sentinel-2 imagery. Fang et al. (2016) found that reflectance at 550 nm was the most sensitive band to estimate flowering coverage with an estimation error below 6% when compared with wavelengths at 490, 670, 720, 800, and 900 nm. Wan et al. (2018) and Gong et al. (2018) reported that combining vegetation index and image classification methods (i.e., k-means clustering method by CIE L*a*b space and pixel-level spectral mixture analysis) improved the accuracy of flower numbers and yield estimation in canola with *R*^2^ values of 0.89 and 0.75, respectively.

Although several studies have detected canola flowering number and predicted yield, most of these field experiments were conducted with relatively few canola lines and environments, which may neglect the effect of genotype and environmental fluctuations on yellowness of flower (Ohmiya, 2011) and petal size (Jiang and Becker, 2003). In addition, yield estimation models used in those studies were based on only one image date (Sulik and Long, 2016; Gong et al., 2018), which ignores the effect of time and duration of flowering (Tayo and Morgan, 1975). Thus, the reflectance data of flowering throughout the flowering period may provide a better estimate of crop yield. Therefore, the objectives of this study were to use UAV multispectral data to detect flowers within a wide range of canola lines and to estimate seed yield in canola using time series imagery collected during the flowering period.

## Materials and Methods

### Experimental Sites and Plant Materials

**Figure 1:**
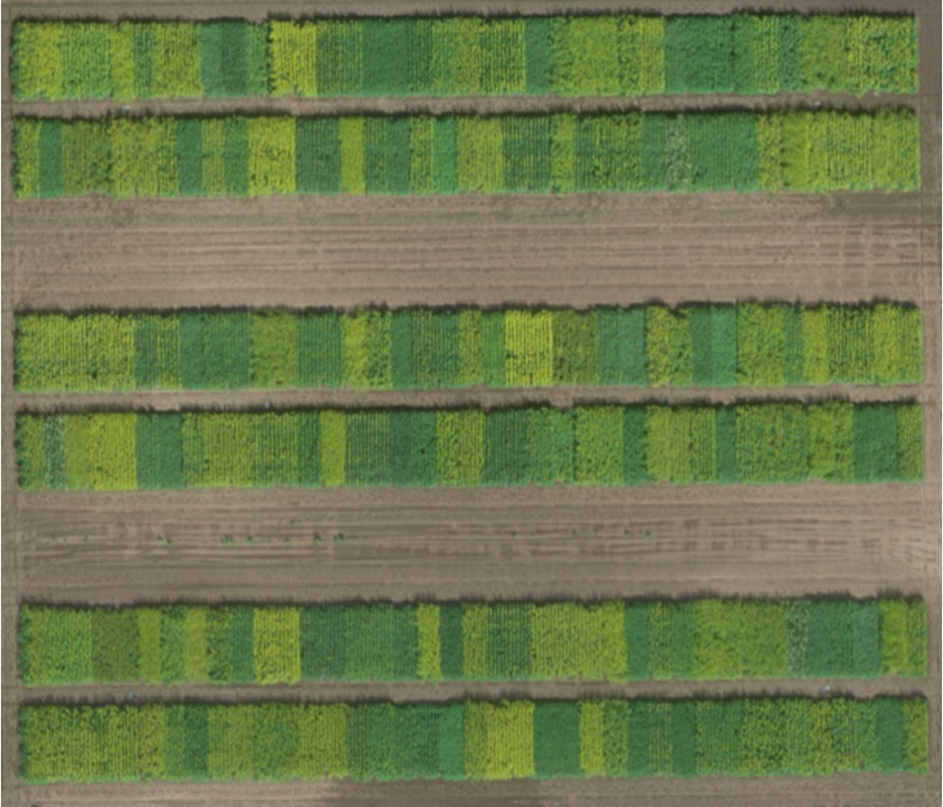
The overview of experimental plot layout at the Agriculture and Agri-Food Canada Research Farm (52° 10' 52.9” N, 106° 30' 10.6” W) near Saskatoon, SK, Canada on July 14, 2016.

The experiment was conducted at the Agriculture and Agri-Food Canada Research Farm near Saskatoon, SK, Canada from 2016 to 2018 (52° 10' 52.9” N, 106° 30' 10.6” W in 2016; 52° 10' 59.3” N, 106° 30' 53.7” W in 2017; and 52° 10' 57.7” N, 106° 30' 01.4” W in 2018), and near Melfort (52° 49' 9.6” N and 104° 35' 46.9” W) and Scott (52° 21' 55.3” N and 108° 52' 32.6” W), SK, Canada in 2017 (Table 1). The soil texture at Saskatoon was a clay loam with a pH of 7.3 and an organic matter content of 5.5%. The field plots were set up in a randomized incomplete block design (rectangular lattice design) with three replications (Figure 1). A rectangular lattice design was used to reduce spatial variability within each block. Individual plot size was 6.0 m long × 1.2 m wide in 2016 and 2018 and 6.0 m long × 1.5 m wide in 2017. Fifty-six genotypes (Saskatoon Research and Development Center, Agriculture and Agri-Food Canada), including 53 diverse *B. napus* lines, two *B. carinata* lines, and a *B. juncea* variety, were selected and planted. Fifty of the diverse lines were used as founders to develop Nested Association Mapping (NAM) population by developing population from crossing to a common reference line (Parkin et al., 2017). This panel, which represents diverse germplasm resources and the historical basis of canola breeding programs, differs in geographic origin, pedigree, phenotypes, and genotype (Parkin et al., 2017). Seeding occurred on May 27, 28, and 21 in 2016, 2017, and 2018, respectively, at a seeding rate of 108 seeds m^−2^ (Table 1). Out of 56 lines, 16 were selected and planted twice in two adjacent but separate plots as double plots. The criteria of line selection for the double plots were based on contrasting seed quality (i.e., seed color, acid detergent lignin, seed glucosinolates, and seed erucic acid) and similarity in flowering timing. The reason for setting double plots was to preserve one plot for imaging without any subsamples being removed. The 16 *B. napus* lines planted in double plots were YN04-C1213, NAM-0, 5, 13, 14, 17, 23, 30, 37, 32, 43, 46, 48, 72, 76, and 79.

**Table 1:** Summary of canola trials and data collection (imagery acquisition and manual flower count) at Saskatoon, SK, Canada from 2016 to 2018 and at Melfort and Scott, SK, Canada in 2017.

**Site**	**Year**	**Seeding date**	**Number of lines/cultivars**	**Flight altitude (m)**	**Image acquisition dates**	**Manual flower count dates**
Saskatoon	2016	May 27	56	20	July 14; 19; 26 August 06	July 15; 22; 29 August 05
	2017	May 28	56	20	July 07; 11; 15; 19; 22; 26 August 01; 09; 16; 22	July 01;18; 25 August 01
	2018	May 21	56	25	June 28 July 06; 09; 16; 20; 24; 27; 30 August 03; 07	July 10; 17; 24; 31
Melfort	2017	May 18	16	15	July 05; 13; 20; 26	July 05; 20; 26
Scott	2017	June 20	16	20	August 09; 16; 29	August 09; 16

The selected 16 *B. napus* lines were planted in a randomized complete block design with three replications at the Melfort and Scott locations in 2017. All lines were planted in 5 m long × 1.2 m wide plots at Melfort and in 5 m long × 1 m wide plots at Scott. Canola was seeded on May 18 at Melfort and June 20 at Scott at a desired seeding rate of 108 seeds m^−2^ (Table 1). Edge^®^ (ethalfluralin) was applied as a pre-emergence herbicide at a rate of 19.1 kg ha^−1^ to control weeds. Any weeds not controlled by the herbicides were removed by hand.

### Image Acquisition

#### Platform and Sensor

The UAV used in this study was a Draganflyer X4-P model in 2016 and 2017 (DraganFly Inc., Saskatoon, SK, Canada). It is a rotary-wing platform with a maximum payload of 800 g. It can semiautomatically depart and land based on GPS navigation mode and optional Surveyor software. Flight mission was planned in Surveyor software (DraganFly Inc., Saskatoon, SK, Canada) by importing ground coordinates of the field boundaries. The other rotary-wing platform was a Draganflyer Commander model (DraganFly, Inc., Saskatoon, SK, Canada), used in 2018, which differs from the X4-P model in its maximum payload capacity (1,000 g).

A multi-spectral camera (RedEdge, MicaSense Inc., Seattle, WA, United States) was used to acquire images (12-bit image) with an image resolution of 1.2 megapixels (1,280 × 960 pixels) for each of five spectral bands (blue: 475 ± 10 nm; green: 560 ± 10 nm; red: 668 ± 5 nm; red edge: 717 ± 5 nm; and near-infrared: 840 ± 20 nm) (Table 2). The focal length of the camera is 5.5 mm and the ground sampling distance at 15, 20, and 25 m above ground level was 1.02, 1.36, and 1.70 cm per pixel, respectively (Table 2). Images of a MicaSense reflectance panel (RedEdge, MicaSense Inc., Seattle, WA, United States) were taken before and after each UAV flight for radiometric calibration. To geo-reference aerial images, six ground control points (GCPs) were distributed across the experimental area during the whole crop season in 2016 at Saskatoon. The size of the GCPs was 60 × 60 cm, which were geolocated by Trimble GeoExplorer 2008 GPS (Trimble Inc., Westminster, CO, United States). GCPs were manually placed at the same location when phenotyping canola by UAV, which provided an overlay of images taken from various dates and reduced workload by using the same geolocation information for each GCP. For the four locations in 2017 and 2018, GCPs were permanently mounted within guard plots to avoid manually carrying GCPs to the field.

**Table 2:** Basic specifications for the multispectral camera (RedEdge) equipped on the unmanned aerial vehicle (UAV) platforms.

**GSD^a^ (cm per pixel) (per band)**	**Flight altitude** **(m)**	**Sensor resolution per band (MP)^b^**	**Focal length (mm)**	**Full width at half maximum (nm)**	**Peak wavelength (nm)**
1.02 1.36 1.70	15 20 25	1.2^c^	5.5	Blue: 465–485 Green: 550–570 Red: 663–673 Red edge: 712–722 NIR: 820–860	Blue: 475 Green: 560 Red: 668 Red edge: 717 NIR: 840

a *GSD: ground sampling distance*.

b *MP: megapixel*.

c *image resolution: 1.2 MP = 1,280 × 960 pixels*.

#### UAV Flight Schedule

The UAV, equipped with a multispectral camera, captured the images of the fields taken weekly during the flowering stage at Saskatoon in 2016 and at Melfort and Scott in 2017 (Table 1). The imagery was collected semiweekly in 2017 and 2018 at Saskatoon for the duration of canola flowering (Table 1). For the Saskatoon location, although weather conditions such as rain, clouds, and heavy wind limited the flight schedule, image timing interval was achieved as close to 7 days in 2016 and to 4 days in 2017 and 2018. For the Melfort and Scott locations in 2017, image collection was carried out at a 7-day interval.

### Image Process and Data Extraction

#### Image Pre-process

Multispectral images were processed, stitched, and calibrated in Pix4Dmapper Pro (Pix4D Inc., San Francisco, CA, United States). Individual images were aligned based on common points from the overlapped images to generate a geo-referenced image that matched the overflown study area. Geometric calibration was done by importing the geo-location of GCPs to reduce geometric distortion problems of the camera. A system coordinate, World Geodetic System 1984, was applied to generate geo-referenced images. The images of the MicaSense reflectance panel were used in the radiometric calibration to enhance spectral consistency between different flight dates. Then, the five generated reflectance maps were exported and used for further analysis.

#### Vegetation Index Calculation, Thresholding, and Integration of Flowering Progress

ArcGIS software version 10.4.1 (ESRI Canada, Toronto, ON, Canada) was applied for plot segmentation, vegetation indices calculation, and thresholding. In this study, the middle three rows for each plot were segmented using polygon shapes with assigned plot numbers. The polygon shapes were generated using the “Create Feature” tool. Vegetation index maps were derived *via* calculation of the reflectance maps using the “Rater calculator” tool. Commonly used vegetation indices, NDVI (Rouse et al., 1974), NDYI (Sulik and Long, 2016), green normalized difference vegetation index (GNDVI) (Gitelson et al., 1996), and normalized difference red edge index (NDRE) (Gitelson and Merzlyak, 1997), were calculated as following equations to compare with the actual flower number counts:

(1) $$NDVI=\left(\frac{R_{NIR}-R_{red}}{R_{NIR}+R_{red}}\right)$$

(2) $$NDYI=\left(\frac{R_{green}-R_{blue}}{R_{green}+R_{blue}}\right)$$

(3) $$GNDVI=\left(\frac{R_{NIR}-R_{green}}{R_{NIR}+R_{green}}\right)$$

(4) $$NDRE=\left(\frac{R_{NIR}-R_{rededge}}{R_{NIR}+R_{rededge}}\right)$$

where R_NIR_, R_red_, R_green_, R_blue_, and R_rededge_ are the reflectance values at bands centered on 840, 668, 560, 475, and 717 nm, respectively (Table 2). NDVI is the most commonly used vegetation index to identify crop growth conditions and yield estimation (Rouse et al., 1974). NDYI has previously shown a strong correlation with seed yield (Sulik and Long, 2016). GNDVI (Gitelson et al., 1996) and NDRE (Gitelson and Merzlyak, 1997) are related to photosynthesis and have been reported in previous research.

Canola flowers and leaf organs co-existed within each plot during flowering; thus, the “Conditional Function” [Con (index map > threshold value, index map, “”)] in the “Raster Calculator” tool was used to separate flowering pixels from non-flowering pixels by applying threshold values on vegetation index maps. Threshold values were manually determined by comparing the composited RGB images with calculated index maps so that most flowering pixels could be selected and segmented. All pixels in the index map that have values larger than the threshold values were kept in a threshold index map, otherwise, pixels were discarded. Then, the “Zonal Statistics” tool was used to extract the summary statistics of the threshold index map, which included the number of flowering pixels per plot.

**Figure 2:**
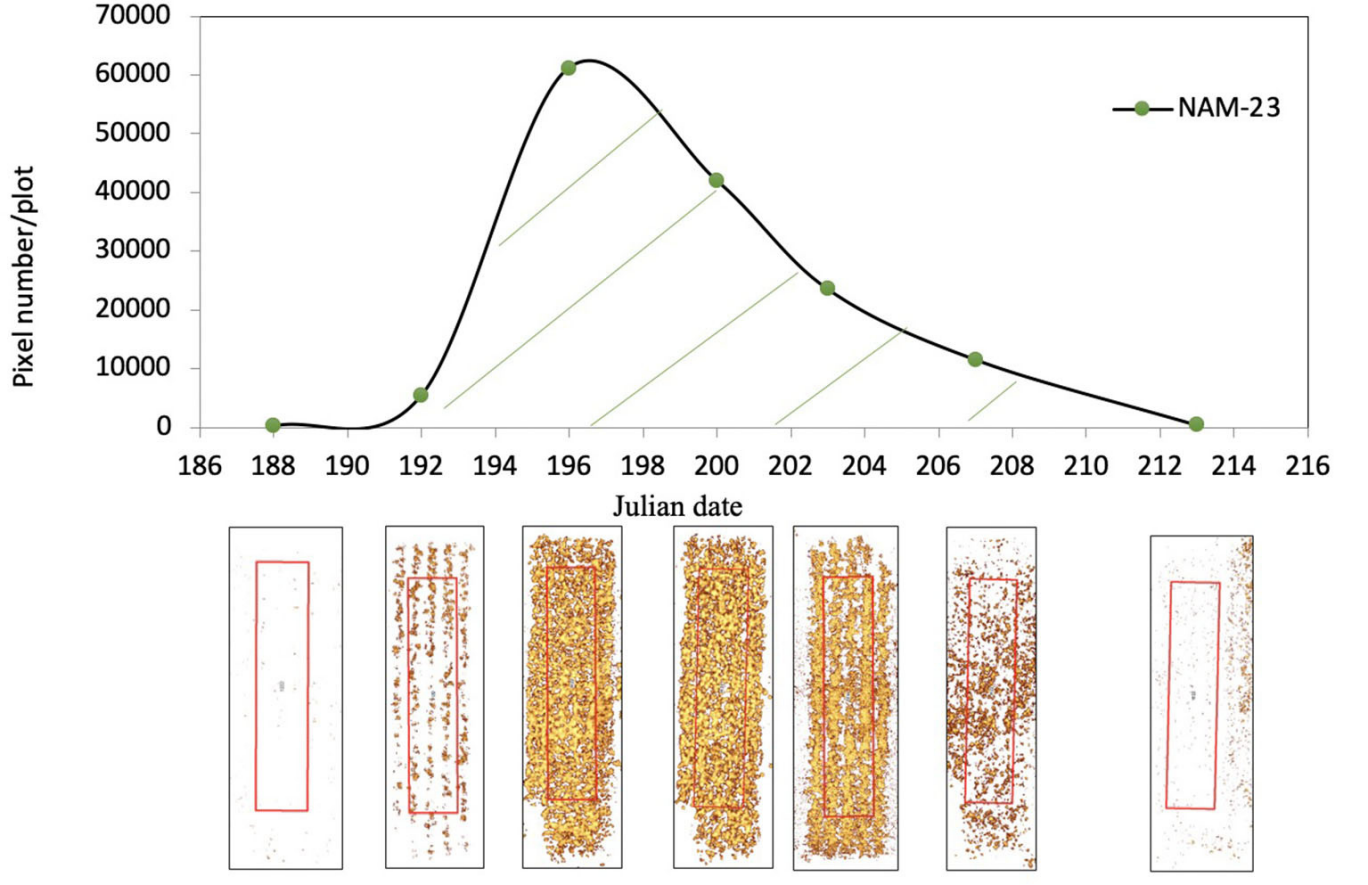
The growth pattern of flowering progress for a *Brassica napus* line (NAM-23) during the flowering stage at the Agriculture and Agri-Food Canada Research Farm (52° 10' 59.3” N, 106° 30' 53.7” W) near Saskatoon, SK, Canada in 2017. The x-axis is the imagery acquisition date (Julian date) in 2017. The y-axis is normalized difference yellowness index (NDYI)-based pixel number per plot. A solid curve line is the flowering progress trend of NAM-23. Seven points on the progress curve line represent NDYI-based pixel number per plot at seven imagery acquisition dates. Seven pictures under each point are corresponding false-color images after thresholding with flowers highlighted in yellow. The region of interest was highlighted in red.

This study involved 56 diverse lines with a high flowering density gradient. It is difficult to determine which image date is proper for yield estimation. For this reason, the area under the flowering progress curve (AUFPC) was used to calculate the integration of flowering progress during the flowering season using the following equation:

(5) $$\begin{array}{l} AUFPC=\left(\frac{F_{1}+F_{2}}{2}-F1\right)\left(t_{2}-t_{1}\right)+\left(\frac{F_{2}+F_{3}}{2}-F1\right)\left(t_{3}-t_{2}\right)\\ \;+\ldots +\left(\frac{F_{n-1}+F_{n}}{2}-F1\right)\left(t_{n}-t_{n-1}\right) \end{array}$$

where F_1_, F_2_, F_3_, F_n−_1, and F_n_ represent the flowering pixel numbers at each image date and t_1_, t_2_, t_3_, t_n−1_, and t_n_ represent Julian date at each image timing. The AUFPC is an adjusted integration equation based on the area under the disease progress curve (AUDPC), which is used in general in pathology studies for estimating the effect of disease progression on crop yield (Jeger and Viljanen-Rollinson, 2001; Simko and Piepho, 2012). Compared with AUDPC, the advantage of the AUFPC is providing a baseline for each line to adjust flowering progress, which can reduce the effect of diverse initial flowering pixel numbers of each line on the calculated area. The AUFPC equation converted several flowering pixel numbers at a series of image timings into a single value for reporting. The larger the AUFPC value is, the further the flowering had progressed. Figure 2 displays an example of flowering progress over time for a line (NAM-23). Seven data points on the curve line represent NDYI-based pixel numbers for each image date. Pictures under the seven points are corresponding threshold index maps. Then, the area under the curve line was calculated using the AUFPC equation (Equation 5) for NAM-23. The same mathematical method was used to calculate flowering progress for all other lines across 5 site years.

### Ground Reference Data/Field Data Collection

The first row of each plot was manually sampled to quantify flowering. Canola flowering typically starts in early July and ends in early August. Flower numbers on the main stem and branches of randomly selected plants were counted at a 7-day interval from July to August. Grain yield was straight combined by a small plot combine harvester when the crop was mature and dry. This occurred multiple times due to differing maturity dates of the *B. napus* lines. To reduce the edge effect, the middle four rows of each plot were harvested. All harvested seeds were air-dried to 10% seed moisture. Final yields were weighed after seed cleaning.

### Statistical Analysis

The PROC LATTICE procedure of SAS version 9.4 (SAS Institute, Cary, NC, United States) was used to analyze the data. The LATTICE procedure reduced variations within blocks. After data adjustment, PROC REG in SAS version 9.4 was used as the statistical tool to investigate the simple linear regressions between ground reference data and imagery. Scatterplots of variables were observed to determine whether data could be combined for analysis. In the case where data could not be combined, data were analyzed within site years.

## Results and Discussion

### Regression Between Flowering Pixel Number and Actual Flower Numbers

These initial results showed that GNDVI and NDRE did not demonstrate significant correlations with the actual flower count (*P* > 0.05, data not shown). Meanwhile, regression results showed that NDYI had greater coefficients of determination (*R*^2^) than NDVI with actual flower numbers within 3 years of study. An increased red light from the yellow petals can reduce NDVI values and affect its ability to detect canola growth conditions. In addition, there was no strong relationship between plot-level NDYI and actual flower numbers in 2016 (data not shown). Noise from soil background and green vegetation within a plot at the early flowering stage may have resulted in these weak relationships. For this reason, we used NDYI maps to extract flowering pixels and remove non-flowering pixels by the thresholding method. We detected and counted flowering pixels when pixel values were greater than NDYI-based threshold levels. Threshold values were 0.59, 0.52, and 0.45 in 2016, 2017, and 2018, respectively.

**Figure 3:**
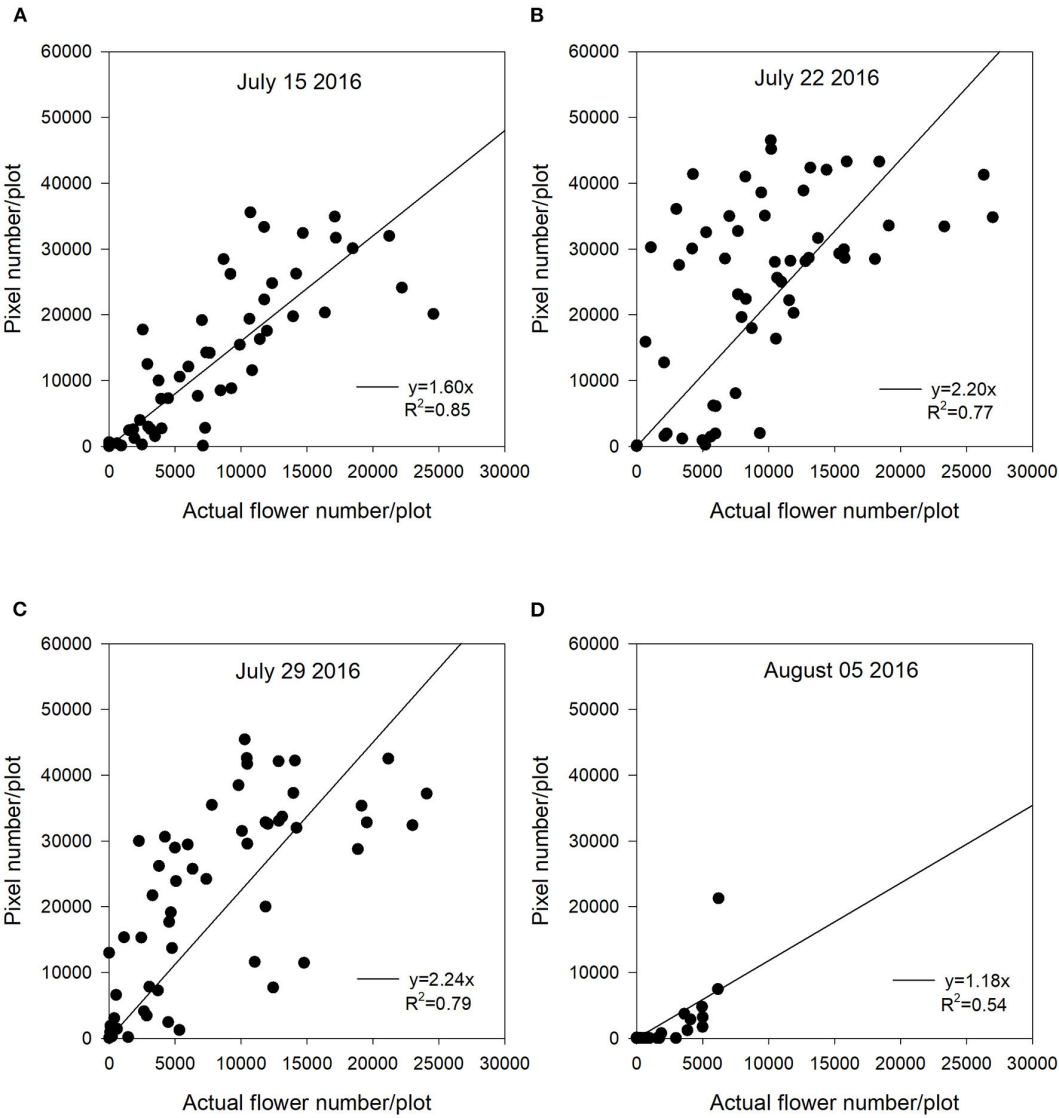
The relationship between actual flower numbers per plot and pixel numbers extracted from aerial images during the flowering stage at Saskatoon, SK, Canada in 2016. Actual flower numbers per plot were manually measured. Pixel number per plot was detected by the thresholding method. **(A)** Regression equation for July 15, 2016: y = 1.60x, *R*^2^ = 0.85. **(B)** Regression equation for July 22, 2016: y = 2.20x, *R*^2^ = 0.77. **(C)** Regression equation for July 29, 2016: y = 2.24x, *R*^2^ = 0.79. **(D)** Regression equation for August 05, 2016: y = 1.18x, *R*^2^ = 0.54.

**Figure 4:**
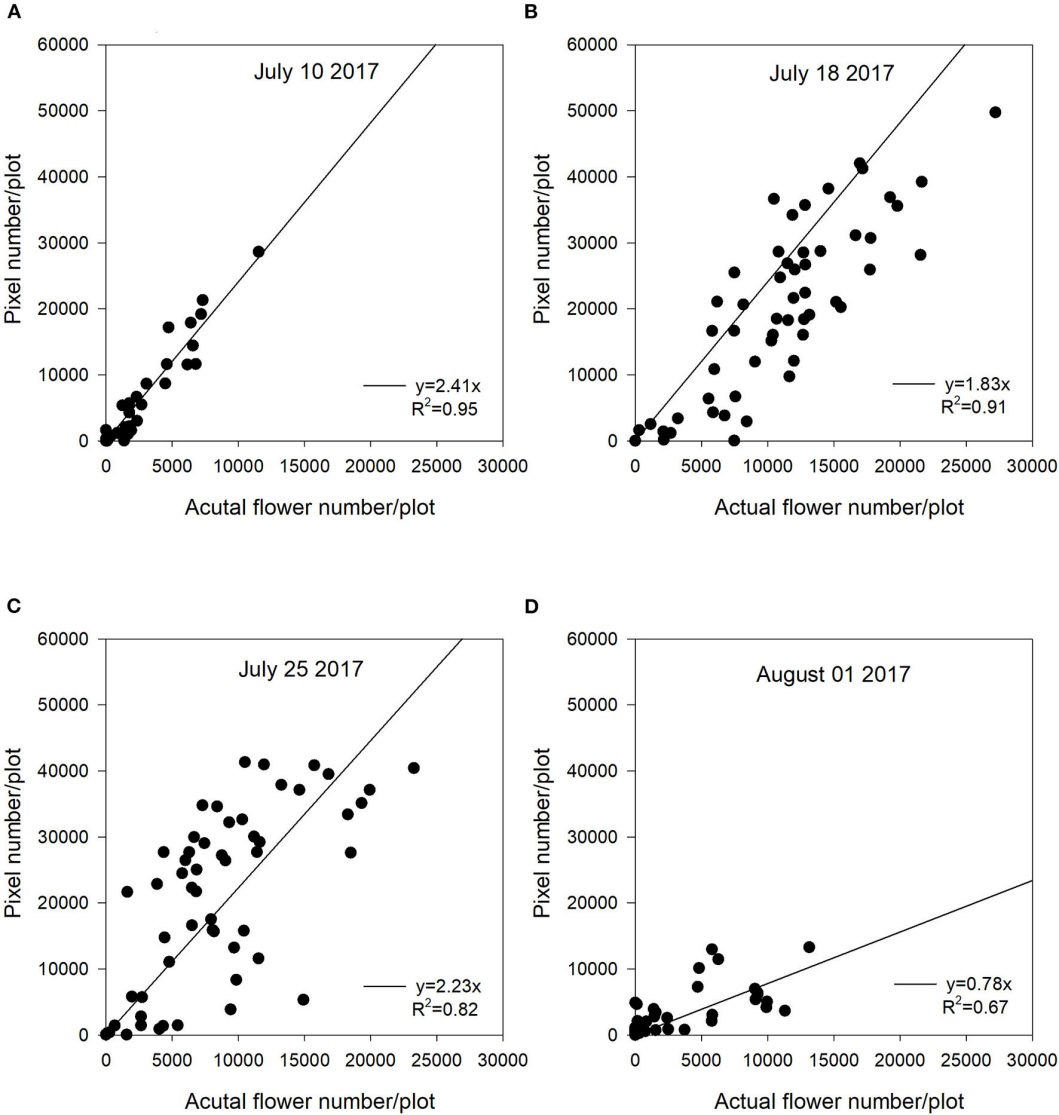
The relationship between actual flower numbers per plot and pixel numbers extracted from aerial images during the flowering stage at Saskatoon, SK, Canada in 2017. Actual flower numbers per plot were manually measured. Pixel number per plot was detected by the thresholding method. **(A)** Regression equation for July 10, 2017: y = 2.41x, *R*^2^ = 0.95. **(B)** Regression equation for July 18, 2017: y = 1.83x, *R*^2^ = 0.91. **(C)** Regression equation for July 25, 2017: y = 2.23x, *R*^2^ = 0.82. **(D)** Regression equation for August 01, 2017: y = 0.78x, *R*^2^ = 0.67.

**Figure 5:**
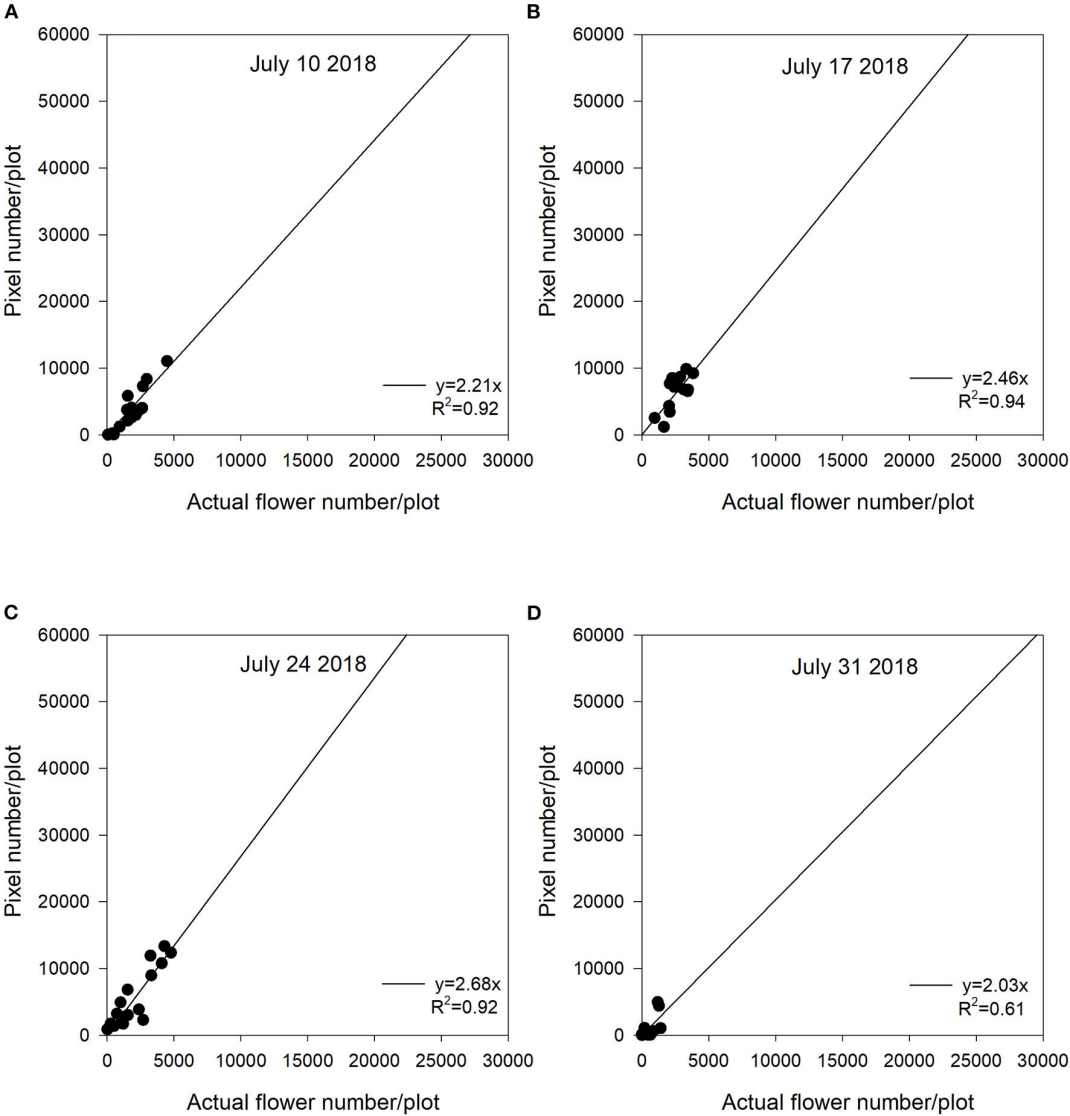
The relationship between actual flower numbers per plot and pixel numbers extracted from aerial images during the flowering stage at Saskatoon, SK, Canada in 2018. Actual flower numbers per plot were manually measured. Pixel number per plot was detected by the thresholding method. **(A)** Regression equation for July 10, 2018: y = 2.21x, *R*^2^ = 0.92. **(B)** Regression equation for July 17, 2018: y = 2.46x, *R*^2^ = 0.94. **(C)** Regression equation for July 24, 2018: y = 2.68x, *R*^2^ = 0.92. **(D)** Regression equation for July 31, 2018: y = 2.03x, *R*^2^ = 0.61.

**Figure 6:**
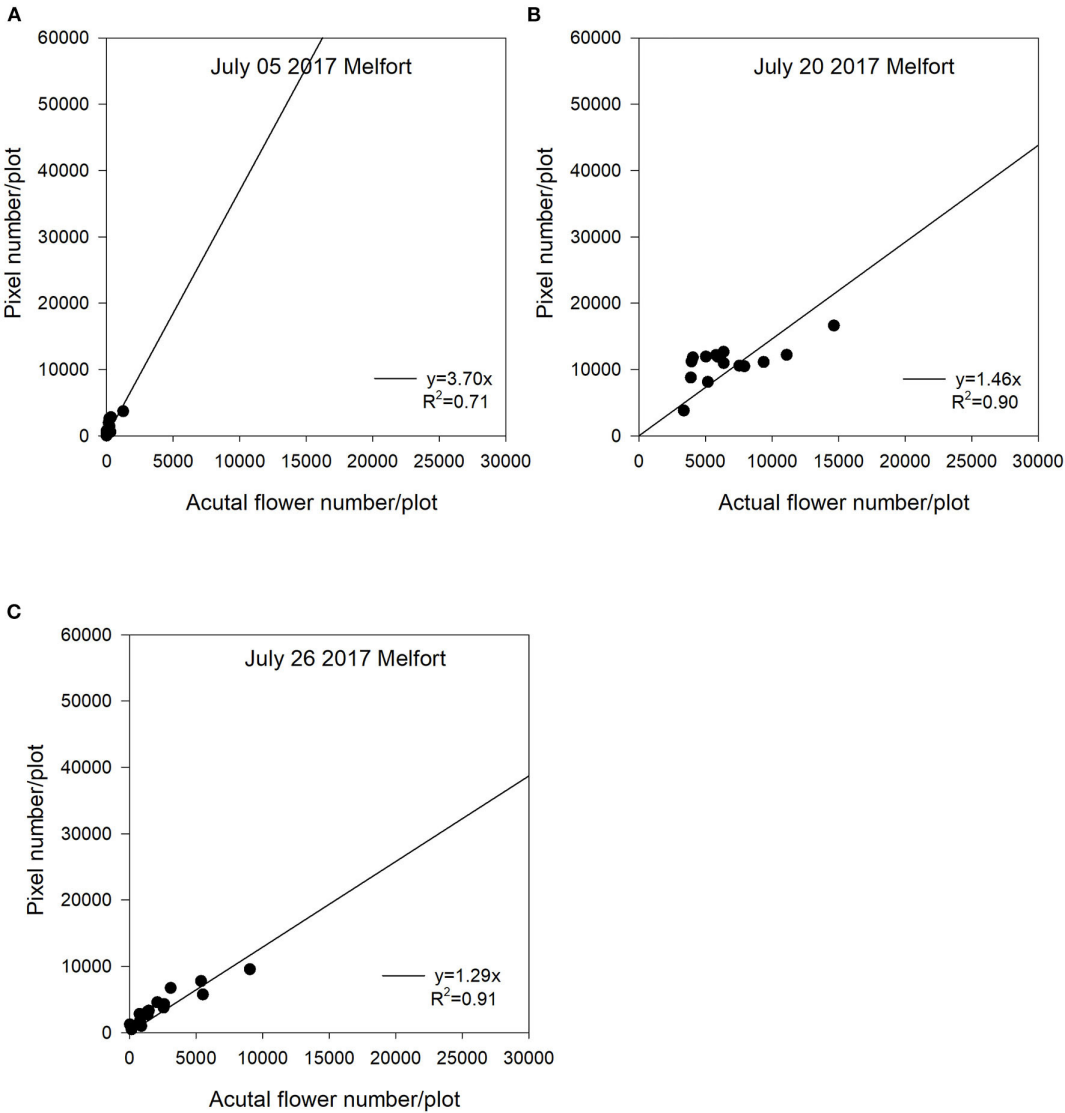
The relationship between actual flower numbers per plot and pixel numbers extracted from aerial images during the flowering stage at Melfort, SK, Canada in 2017. Actual flower numbers per plot were manually measured. Pixel number per plot was detected by the thresholding method. **(A)** Regression equation for July 05, 2017: y = 3.70x, *R*^2^ = 0.71. **(B)** Regression equation for July 20, 2017: y = 1.46x, *R*^2^ = 0.90. **(C)** Regression equation for July 26, 2017: y = 1.29x, *R*^2^ = 0.91.

**Figure 7:**
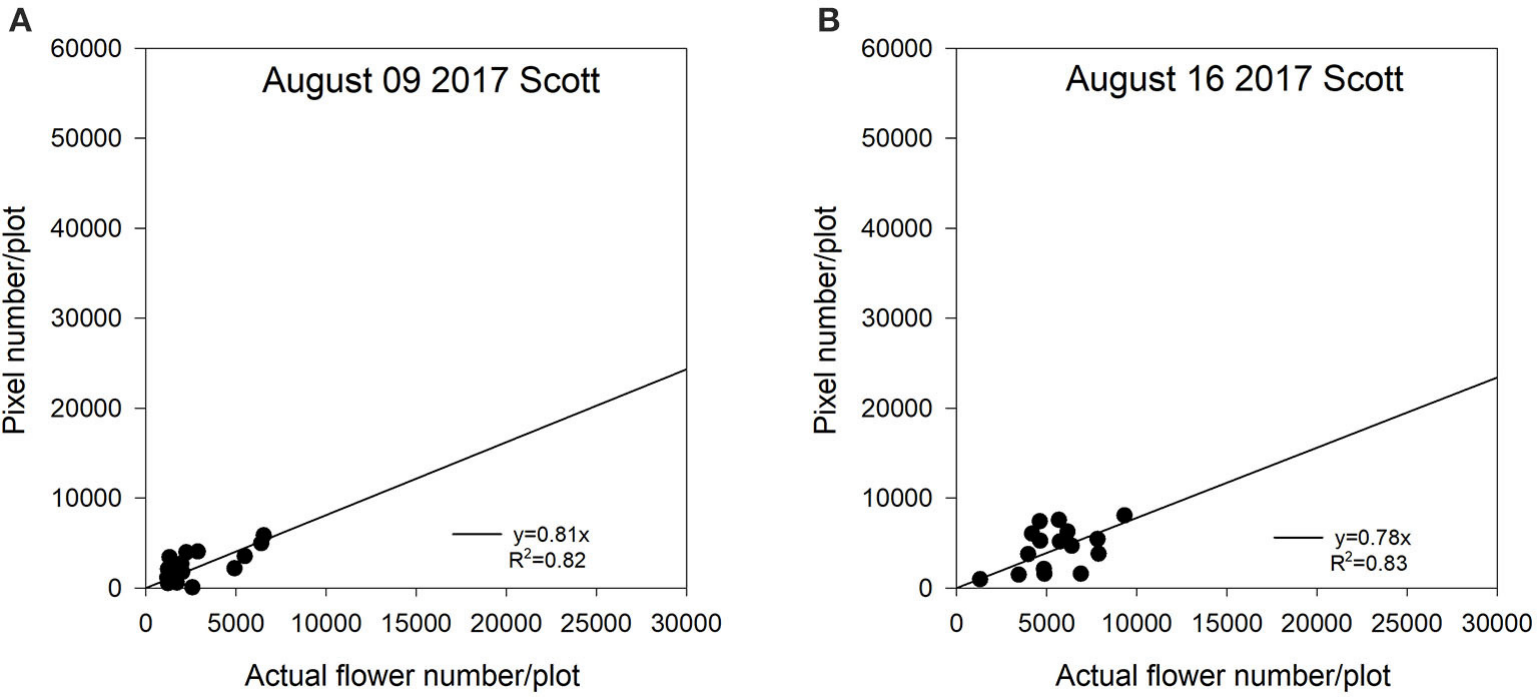
The relationship between actual flower numbers per plot and pixel numbers extracted from aerial images during the flowering stage at Scott, SK, Canada in 2017. Actual flower numbers per plot were manually measured. Pixel number per plot was detected by the thresholding method. **(A)** Regression equation for August 09, 2017: y = 0.81x, *R*^2^ = 0.82. **(B)** Regression equation for August 16, 2017: y = 0.78x, *R*^2^ = 0.83.

Across 5 site years, the *R*^2^ values between flowering pixel numbers and actual flower numbers ranged from 0.54 to 0.95 during flowering duration (Figures 3–**7**). There were significant relationships between flowering pixel numbers and actual flower numbers in 2016 at Saskatoon (Figure 3). Not surprisingly, the early flowering stage (July 15) had the strongest regression relationship with actual flower numbers with an *R*^2^ of 0.85 (Figure 3A). Developing flowers were on the upper part of a plant at the early flowering stage so sensors could easily detect these early-blooming flowers. Whereas, the late flowering stages (August 05) showed the weakest regression (*R*^2^ = 0.54) (Figure 3D), which may be a result of the lower sensitivity of NDYI to differentiate yellow flowers and dark green pods. Dark green pods impart more green reflectance, which can make NDYI less sensitive to yellow flowers, as yellow is a composite color of green and red (Yates and Steven, 1987; Sulik and Long, 2015, 2016). Additionally, the potential reason why it had the smallest *R*^2^ value is that many flowers growing on the lower branches adversely affected the ability of the sensor to detect the late-developing flowers.

The Saskatoon location in 2017 and 2018 had similar regression patterns between flowering pixel numbers and actual flower numbers (Figures 4, 5). There were very strong relationships at the early flowering stages (July 10, 2017 and July 17, 2018) (Figures 4, 5). Similar to 2016, the relationships became weaker with the late flowering stages (August 01, 2017 and July 31, 2018) (Figures 4, 5). Although the late flowering stages had weaker regressions compared with the early flowering timing, the regressions at the peak flowering dates (July 25, 2017 and July 24, 2018) were relatively strong (Figures 4, 5).

For the Melfort location in 2017, the first image date (July 05) had the weakest regression (*R*^2^ = 0.71) (Figure 6A). Variability from subsampling plants can be a potential reason for decreased regressions at the early flowering stage. However, the peak flowering time (July 20) and late flowering stage (July 26) showed strong relationships with the value of *R*^2^ of up to 0.91 (Figures 6B,C). The potential reason why this site year had a greater *R*^2^ at the late flowering stage is that flight altitude (15 m) at Melfort in 2017 was lower than the other site years (Table 1). The high resolution may have increased the ability of the sensor to detect flowers growing lower in the canopy. Although the flight altitude was relatively low compared with other locations, there was no significant canopy movement due to the UAV platform. The seeding date at Scott was June 22, 2017. Flowering started relatively late with a shorter duration compared with other site years. There was no imagery collected at the end of the flowering stage, and thus, those relationships are unknown. At Scott, the *R*^2^ values for the regressions between flowering pixel numbers and actual flower numbers followed similar patterns as the Saskatoon location. The early flowering stage (August 09) and the peak flowering time (August 16) had strong relationships (Figures 7A,B).

In this study, we used a zero-intercept linear regression model in the regression analysis as there was no flowering pixel prior to the commencement of flowering. Furthermore, the fitted intercept values were close to zero in most cases. For the Saskatoon location over 3 years, slopes were relatively consistent at the early flowering stages (Figures 3–5). Slope values became smaller with the delayed flowering stage. There was a smaller slope value at the late flowering stage (slope = 1.18) compared with the peak flowering time (slope = 2.20) at Saskatoon in 2016. The Saskatoon location in 2017 and 2018 had similar patterns (Figures 4, 5). The Melfort location had similar patterns with a smaller slope at the late flowering stage (Figure 6), but the slope of the first image date (slope = 3.70) was greater than the other image dates. This indicated that early flowering imagery overestimated the actual flower numbers. Experimental plots at this location showed non-uniform flowering with fewer flowers at the front of each plot, which may be caused by the edge effect. Thus, manual flower count based on subsampling plants at the front row of a plot may not accurately represent the average flower numbers. In 2017, at Scott, slopes were consistent at the early and the peak flowering times (Figure 7). The slope values at this location were smaller than the other site years. A potential reason for this underestimation of flower numbers is that the plots had a more condensed canopy and there were more branches at this site year than other site years (data not shown) due to poor emergence percentage. Thus, for the Scott location, there were more flowers produced on the lower branches which could not be detected by the sensor. As mentioned above, there was no available data collected at the end of flowering; thus, the relationship at this stage is unknown.

In general, although the linear regression slopes varied across site years, the high *R*^2^ values indicated that the flowering pixel numbers extracted from the threshold NDYI map performed well to predict actual flower numbers at the early and peak flowering stages in canola (*R*^2^ up to 0.95). These results were consistent with that reported by Sulik and Long (2015), wherein the ratio of blue and green strongly correlated with the yellow flowers in canola with a significant *R*^2^ value of 0.87 at the full flowering stage. Wan et al. (2018) reported good estimation for the flowering number of canola using the k-means clustering method based on the CIE L*a*b space model during the full flowering period. Xu et al. (2018) found that white cotton flowers had higher prediction accuracy at the early flowering stage. The lower classification accuracy at the later growth stage may have resulted from coverage of leaves which increased misclassified non-flowers when using a convolutional neural network (Xu et al., 2018). They recommended that using one raw image might solve this issue, as more cotton flowers would be detected from different perspectives. Moreover, the early flowering stages across 5 site years showed greater slope values, as most flowers at this early stage were visible and had less overlap. In contrast, flowers growing on lower branches were likely to be underestimated at the late flowering stages. Subsampling variability may make the actual flower count non-representative for a plot, which may reduce the accuracy of flower estimation.

### Yield Estimation Using Integrated Flowering Accumulation During Flowering Period

Flowering pixel numbers derived from the threshold NDYI map were able to estimate actual flower numbers across 5 experimental site years. Initially, we did regression analysis between yield and flowering pixel numbers at each image date. Among the 5 site years, in most cases, there were no significant relationships until the middle of July when most varieties started blooming (Table 3). In addition, it is difficult to determine a single well-defined image time for crop yield estimation because of various environmental fluctuations and various flowering timings in large-scale breeding programs, especially involving many diverse lines. Furthermore, we may miss important flowering progress information if yield estimation is only based on the imagery from a single date (Haynes and Weingartner, 2004; Gan et al., 2016). Although flower formation at the later stage may contribute less than early timing points, they may still have the potential to increase final grain yield. Therefore, integrating all aspects of the entire flowering duration using AUFPC can reflect flowering accumulation progress and improve the accuracy of crop yield estimation.

**Table 3:** The coefficient of determination (*R*^2^) between flowering pixel numbers from a single image date and yield at Saskatoon, SK, Canada from 2016 to 2018 and at Melfort and Scott, SK, Canada in 2017.

**Site**	**2016**	***R*^**2**^**	**2017**	***R*^**2**^**	**2018**	***R*^**2**^**
Saskatoon	July 14	0.04	July 07	0.02	June 28	<0.01
	July 19	<0.01	July 11	<0.01	July 06	0.02
	July 26	0.02	July 15	0.04	July 09	0.06
	August 06	0.04	July 19	0.29^***^	July 16	0.36^***^
			July 22	0.33^***^	July 20	0.22^***^
			July 26	0.06	July 24	0.07
			August 01	0.06	July 27	<0.01
			August 09	0.02	July 30	0.03
			August 16	0.05	August 03	0.03
			August 22	0.05	August 07	0.02
Melfort			July 05	<0.01		
			July 13	0.23^*^		
			July 20	0.02		
			July 26	0.14		
Scott			August 09	0.46^**^		
			August 16	0.32^*^		
			August 29	0.01		

* *Significant at the 0.05 probability level*.

** *Significant at the 0.01 probability level*.

*** *Significant at the 0.001 probability level*.

**Figure 8:**
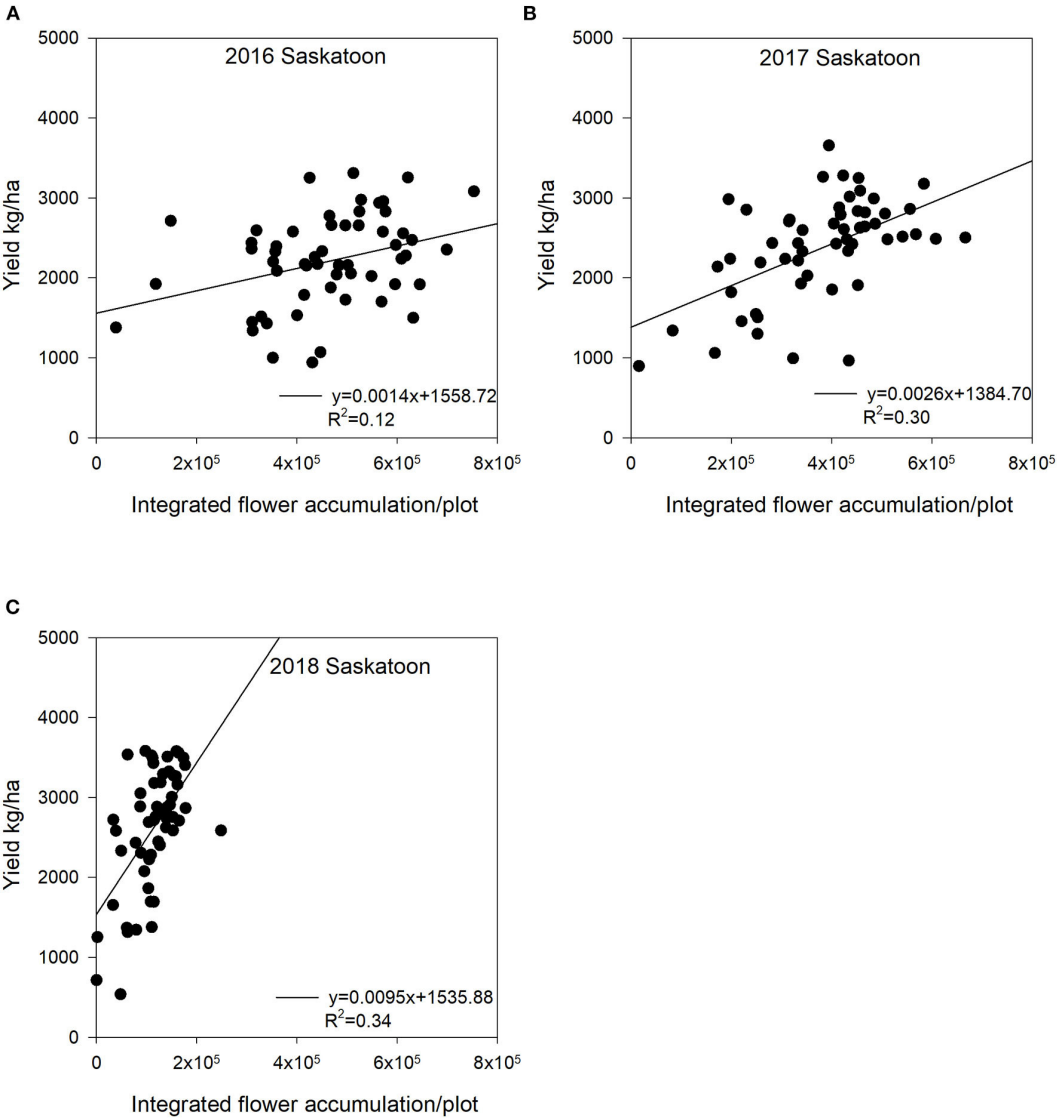
The relationship between seed yield and integrated flower accumulation at Saskatoon, SK, Canada from 2016 to 2018. The integrated flower accumulation was calculated using the area under the flowering progress curve function. **(A)** Regression for the Saskatoon location in 2016: y = 0.0014x + 1558.72, *R*^2^ = 0.12. **(B)** Regression for the Saskatoon location in 2017: y = 0.0026x + 1384.70, *R*^2^ = 0.30. **(C)** Regression for the Saskatoon location in 2018: y = 0.0095x + 1535.88, *R*^2^ = 0.34.

**Figure 9:**
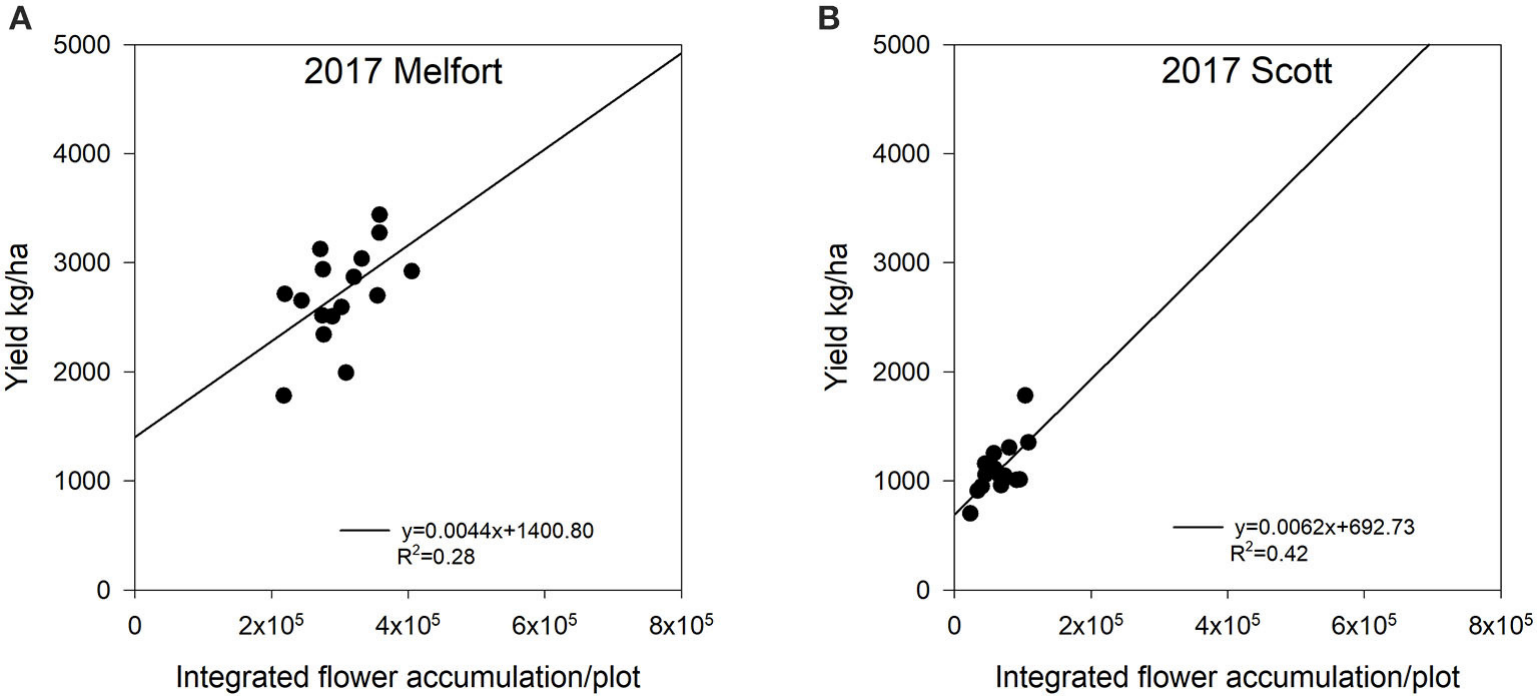
The relationship between seed yield and integrated flower accumulation at Melfort and Scott, SK, Canada in 2017. The integrated flower accumulation was calculated using the area under the flowering progress curve function. **(A)** Regression for the Melfort location in 2017: y = 0.0044x + 1400.80, *R*^2^ = 0.28. **(B)** Regression for the Scott location in 2017: y = 0.0062x + 692.73, *R*^2^ = 0.42.

We found significant relationships between integrated flower accumulation and yield during the flowering period (Figures 8, 9). In 2016, at Saskatoon, integrated flower accumulation had a moderate relationship with yield (*R*^2^ = 0.12, *P* < 0.05) (Figure 8A). We calculated the flower accumulation progress by integrating the flowering pixel numbers over four image dates at a 7-day interval, which missed the starting point of the flowering period. There was no adequate imagery data for the entire flowering period, so it may be the reason for the low accuracy of yield estimation. In both 2017 and 2018 at Saskatoon, we collected imagery semiweekly (Table 1). For the 2 site years, the relationships between integrated flower accumulation and seed yield were relatively stronger compared to the 1st experimental year (*R*^2^ = 0.30, *P* < 0.05 in 2017; *R*^2^ = 0.34, *P* < 0.05 in 2018) (Figures 8B,C). At the Melfort and Scott locations in 2017, there were more consistent and stronger regressions (Figure 9) using the integration of flowering progress, when compared with a single image date (Table 3).

In general, compared with using a single image, applying the integration of flowering progress to estimate yield includes more information to provide consistent accuracy (Figures 8, 9). Although the *R*^2^ values for yield estimation are not very high, our results still demonstrate potential ability of AUFPC to predict yield, especially for crops producing bright flowers (e.g., canola and cotton) under different environmental conditions.

Several studies have reported similar results (Sulik and Long, 2016; Gong et al., 2018; Xu et al., 2018; Hassan et al., 2019). Sulik and Long (2016) reported that the plot-level NDYI values during flowering had high accordance with field yield observations (*R*^2^ = 0.72), which showed a better correlation with seed yield than NDVI at the peak flowering time in canola. Gong et al. (2018) found that NDVI multiplied by leaf-related canopy fraction had the strongest relationship with canola yield with low estimation errors (coefficient of variation < 13%) at the early flowering stages. Some research also investigated yield estimation using canopy reflectance data in other crops including cotton and wheat (Xu et al., 2018; Hassan et al., 2019). Xu et al. (2018) reported that the estimated cotton flower numbers derived from aerial images using a convolutional neural network significantly correlated with cotton yield (*R*^2^ = 0.36). Hassan et al. (2019) reported that UAV-based NDVI measured at the grain filling stage could be a promising tool for wheat yield prediction with *R*^2^ ranging from 0.83 to 0.89 in field conditions.

Our regression results had smaller *R*^2^ values compared with the previous studies. This is probably associated with many diverse lines (i.e., 56 diverse lines) estimated in this study. Most previous research only planted one or few lines. The stability of pigments in rapeseed flowers for each line may change under different developmental stages (Ohmiya, 2011). These factors can impact yellow to some degree (Ohmiya, 2011). The inconsistent yellowness may explain that the more varieties included in regression analysis, the less model variation could be explained by integrated flower accumulation. Furthermore, flowering pixels extracted based on threshold values may not be highly consistent over the flowering stage, as each threshold value was determined manually. In addition, canola yield components include plant density, pod number per plant, seed number per pod, and seed weight. Although pod number per plant is highly correlated with seed yield (Tayo and Morgan, 1975; McGregor, 1981; Ivanovska et al., 2007), only 45% of flowers produce pods (McGregor, 1981). Seed weight per pod and thousand seed weight also significantly correlated with seed yield (Ivanovska et al., 2007). The simple regression analysis of flower numbers could not fully explain yield variation. Additional yield components considered in the yield estimation model would improve the accuracy of seed yield estimation. Finally, flower abortion and poor pod formation can happen under drought and heat stress during the crop season (Faraji et al., 2008). Flowering progress only reveals part of crop growth stages, so some varieties even with high AUFPC may end up with low yield under stress, which may result in a weaker relationship between integrated flower accumulation and seed yield. Combining UAV-based reflectance data at both flowering and pod stages may enhance yield estimation accuracy.

Usually, breeding programs need to assess a large number of varieties or breeding lines across multiple environmental conditions. Therefore, from a practical perspective, these results revealed a more realistic yield estimation trend for large-scale breeding programs. Moreover, most previous research used one image date or selected the largest reflectance index value for each plot across all sampling dates to estimate crop yield. In fact, it is difficult to determine the best image date for yield estimation using multiple crop varieties grown in differing environmental conditions. Fluctuating environments can influence flowering progress; therefore, integrated flower accumulation is a promising and predictable variable in the descriptive yield model.

## Conclusions

In this study, we proposed a simple and effective approach to estimate relative flower numbers and model seed yield based on the integrated flowering pixel. This study results showed that flowering pixel numbers estimated by the thresholding method regressed strongly with manual flower count during the flowering stage with an *R*^2^ value of up to 0.95, indicating that flowering pixel numbers can be used as a good indicator of flowering intensity in the field. Additionally, the integrating flowering progress from consecutive images *via* AUFPC math function was more consistently and strongly related to yield compared with using a single image date because integrated flowering pixel over time utilizes more growth information. Therefore, the integrated flower accumulation can be a good indicator for yield estimation. These tools do not require extra coding or strong computer science background, can be used for calculating thresholding and vegetation indices, and is a convenient tool for agronomists and breeders. Future studies need to consider and test a multivariate model including multiple vegetation indices related to other yield components and more reflectance information from the pod stage to improve yield estimation accuracy.

## Data Availability Statement

The raw data supporting the conclusions of this article will be made available by the authors, without undue reservation.

## Author Contributions

SS and SV designed the field experiments. SV and IP provided and prepared the plant materials. SS contributed to funding acquisition. TZ, HD, and SS collected the images and performed imagery pre-processing. TZ conducted ground data collection, performed the statistical analysis, and wrote the manuscript under the supervision of SS and with contributions from SV, HD, EJ, and XG. All authors contributed to the article and approved the submitted version.

## Conflict of Interest

The authors declare that the research was conducted in the absence of any commercial or financial relationships that could be construed as a potential conflict of interest.
